# Evolution of Two Major Zika Virus Lineages: Implications for Pathology, Immune Response, and Vaccine Development

**DOI:** 10.3389/fimmu.2018.01640

**Published:** 2018-07-18

**Authors:** Jacob T. Beaver, Nadia Lelutiu, Rumi Habib, Ioanna Skountzou

**Affiliations:** Department of Microbiology and Immunology, Emory Vaccine Center, Emory University School of Medicine, Atlanta, GA, United States

**Keywords:** Zika virus, evolution, transmission, tissue tropism, phylogeny, immunology, vaccines

## Abstract

Zika virus (ZIKV) became a public health emergency of global concern in 2015 due to its rapid expansion from French Polynesia to Brazil, spreading quickly throughout the Americas. Its unexpected correlation to neurological impairments and defects, now known as congenital Zika syndrome, brought on an urgency to characterize the pathology and develop safe, effective vaccines. ZIKV genetic analyses have identified two major lineages, Asian and African, which have undergone substantial changes during the past 50 years. Although ZIKV infections have been circulating throughout Africa and Asia for the later part of the 20th century, the symptoms were mild and not associated with serious pathology until now. ZIKV evolution also took the form of novel modes of transmission, including maternal–fetal transmission, sexual transmission, and transmission through the eye. The African and Asian lineages have demonstrated differential pathogenesis and molecular responses *in vitro* and *in vivo*. The limited number of human infections prior to the 21st century restricted ZIKV research to *in vitro* studies, but current animal studies utilize mice deficient in type I interferon (IFN) signaling in order to invoke enhanced viral pathogenesis. This review examines ZIKV strain differences from an evolutionary perspective, discussing how these differentially impact pathogenesis *via* host immune responses that modulate IFN signaling, and how these differential effects dictate the future of ZIKV vaccine candidates.

## Introduction

Zika virus (ZIKV) has garnered international attention due to its rapid worldwide expansion since 2015 when an epidemic struck Brazil, resulting in a newly identified pathology including severe neurological impairments such as microcephaly, which is now part of the congenital ZIKV syndrome, as well as Guillain–Barré syndrome (GBS) afflicting adults (1). The World Health Organization declared ZIKV a Public Health Emergency of International Concern in February 2016, during which time ZIKV was spreading rapidly across South America, the Caribbean, and into the United States (1). This precarious outbreak in Brazil spread rampant across the western continents raising critical questions pertaining to the evolution of this virus. Prior to 2015, ZIKV infections were limited geographically to Africa and Asia and were reported to be asymptomatic, and approximately 20% mildly symptomatic represented as a self-limiting febrile illness with most common symptoms maculopapular rash, conjunctivitis, and joint pain (2). The mounting evidence that ZIKV is now causing neuropathology and fetal brain disruption, as well as rising concerns over novel modes of ZIKV transmission suggests an evolutionary change in the molecular and genetic structure of ZIKV strains that has contributed to its rapid expansion, severity of pathogenicity, and multiple routes of infections. These increasing adverse effects depicts why an analysis of the phenotypic differences between the African and Asian lineages, as well as between the many strains, which have evolved under each branch, is a vital component of our ongoing effort to develop vaccines or therapeutics and fill major gaps of knowledge regarding ZIKV pathogenesis.

## History of Virus Emergence

Zika virus was discovered in the Zika Forest of Uganda in 1947 by Alexander Haddow and George Dick during a surveillance investigation of yellow fever in rhesus macaques in Uganda (3). The virus was later isolated from the *Aedes africanus* mosquito collected at the same site (4). The first human case occurred in Nigeria in 1954, but it was not until 1966 that ZIKV was first detected in Asia alongside the first evidence of transmission by an urban vector, *Aedes aegypti* mosquitoes from Malaysia (4, 5). We know that two major lineages of ZIKV were formed at this time, African and Asian, which is confirmed by current genetic and phylogenetic analyses (2). ZIKV made no headlines until an outbreak in 2007 on Yap Island, Micronesia, rendering 73% of the residents infected (6). Despite the presence of DENV IgM in all affected individuals, the unique symptomatic presentation was definitively identified as ZIKV-induced. The next outbreak was 6 years later in French Polynesia, spreading to several other islands in Oceania. The most commonly reported symptoms in the Yap Island and French Polynesian outbreaks included rash, fever, arthralgia, and non-purulent conjunctivitis (7, 8). However, the first case with GBS as a complication of ZIKV infection was reported in the 2013 outbreak in French Polynesia (7). It was also in 2013 when it was discovered that ZIKV transmission could occur through blood or other bodily fluids and not just through mosquito bites.

Brazil was the next location to experience an outbreak early in 2015. Phylogenetic and molecular clock analyses revealed that there was a single introduction of ZIKV to the country (9). The virus was likely brought to Brazil by a traveler from French Polynesia after a stop at Easter Island (10–12). A recent article from Passos et al. performed a retrospective blood blank analysis on 210 samples collected from patients during a DENV-4 outbreak that occurred in early 2013 (13). Of these samples, 10% showed a singly positive qRT-PCR result for ZIKV, and only 2% demonstrated consistently positive results across triplicate samples. While the cycle threshold for positive results by this group is less stringent than those of other groups, it regardless provides potential insight that ZIKV may have been present in South American countries as early as April of 2013.

From the confirmed 2015 cases, it took less than 1 year for the virus to spread throughout Brazil, into neighboring South American countries, and into Central and North America. The increase in GBS cases was reported in Brazil, Colombia, Suriname, and Venezuela and microcephaly cases in NE Brazil, which included neurological disorders and neonatal malformations (12, 14–23). The remarkable rise of infants born with microcephaly in Brazil set off international alarms and garnered global attention (24). Sequence homology studies reveal that of the two ZIKV lineages, the strains responsible for the human outbreaks throughout the Americas were phylogenetically closest to the Asian lineage (25).

## Molecular Biology of ZIKV

### ZIKV Genome Organization

Zika virus is a positive single-stranded RNA virus that belongs in the *Flaviviridae* family. This family includes the human pathogenic viruses, Japanese encephalitis virus, dengue virus type 1–4 (DENV), yellow fever virus (YFV), West Nile virus (WNV), and tick-borne encephalitis virus. To better understand how ZIKV might be evolving, it is important to understand its genomic structure. The genome is a 10.8 kb single-strand, positive-sense RNA molecule that consists of a 5′ untranslated region (UTR) (~107 nt), one open reading frame (ORF) (~10.2 kb), and a 3′ UTR (~420 nt). The ORF encodes a polyprotein precursor that is processed into three structural proteins; capsid (C), pre-membrane/membrane (prM), and envelope protein (E) as well as seven non-structural proteins (NS1, NS2A, NS2B, NS3, NS4A, NS4B, and NS5). The viral polyprotein is co-translationally or co-post-translationally cleaved by viral NS2/NS3 protease, host signal peptidase (C/prM, prM/E, E/NS1, 2K/NS4B) and a host protease (NS1/NS2A). The pr- fragment of the prM protein is cleaved by furin in the trans-Golgi apparatus to generate mature virions. The major surface glycoprotein involved in host cell binding and membrane fusion is E protein. Viral reproduction is accomplished through the non-structural proteins (NS1-NS5), which serve as self-cleaving peptidases, along with the viral RNA-dependent RNA-polymerase. The genome organization and major protein functions of ZIKV are highly similar to all other members in the *Flavivirus* genus (25–27).

As RNA genome viruses are strategically organized to contain the minimal number of genes required for sufficient replication and host immune evasion, many RNA viruses have evolved innovative methods for manipulating subverting molecules within their host cells (28). Among these are non-coding, subgenomic RNAs. These subgenomic flavivirus RNA components (sfRNA) have been implicated in both the reduction of type I interferon (IFN) transcription, and in mediating resistance to cellular exonucleases that would degrade genomic transcripts, such as Xrn1 (29, 30). While the complete functional role of sfRNAs remains unknown, few key pieces of information have already emerged regarding ZIKV. Of these, work by Donald et al. suggests that sfRNA of ZIKV can not only inhibit the type I IFN response by means of a pseudo-knot tertiary structure, but may do so in a manner that is more broad than those of other flaviviruses, such as DENV (31). Additionally, the difference in ZIKV lineage does not impact the generation of these sfRNAs, and is unlikely to impact the predicted tertiary structure.

### Genetic Evolution of the Virus

**Figure 1:**
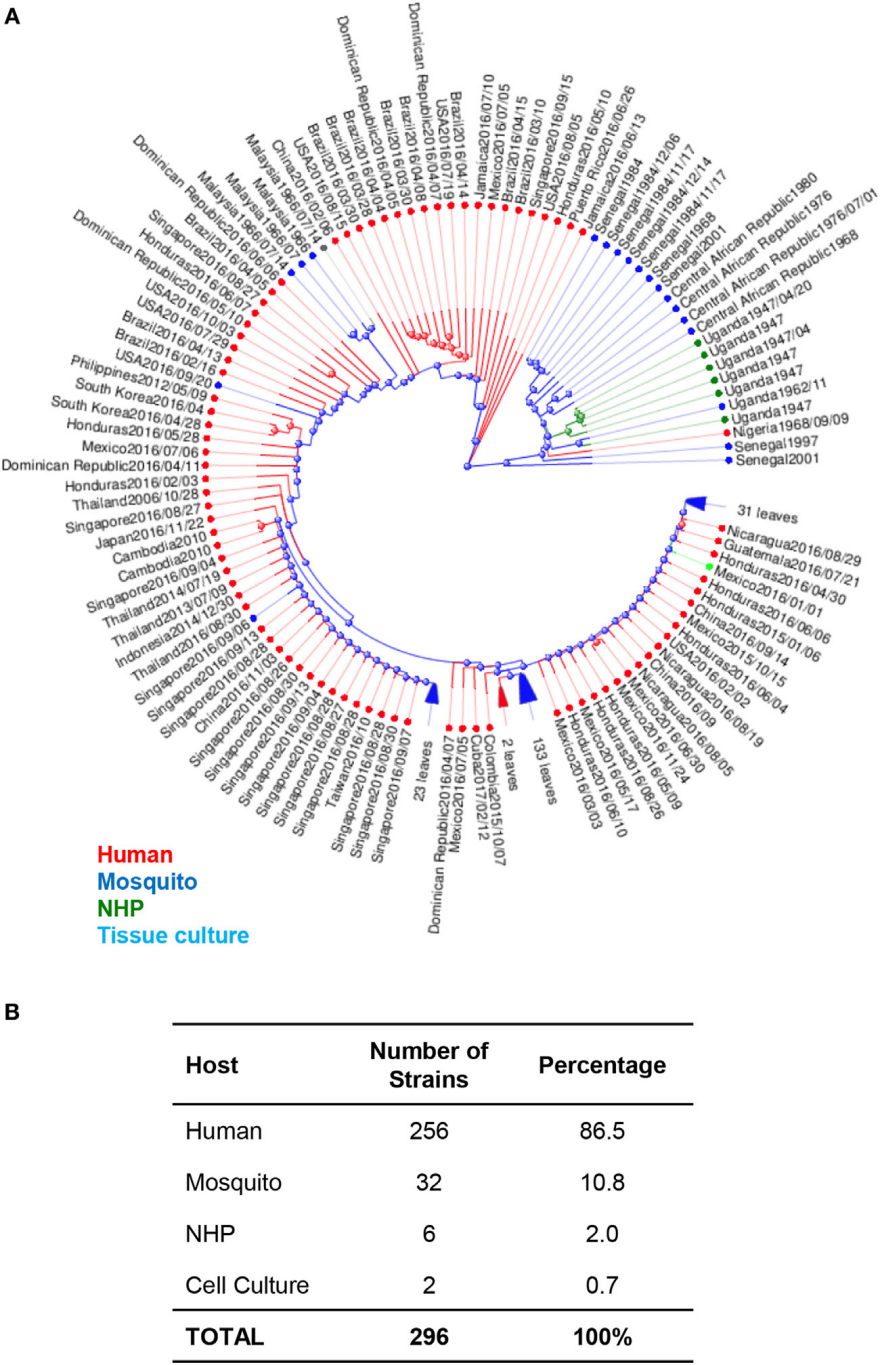
Phylogenetic analysis of Zika virus (ZIKV) genomes by host. **(A)** Phylogenetic analysis of 296 available ZIKV genomes was organized according to the host species of isolation using Virus Variation analysis system available through NCBI. Only complete nucleotide genomes were screened and duplicate strains were removed to produce 296 unique strains. Strains isolated from humans, mosquitos, NHPs, and cell cultures are labeled with red (humans), blue (mosquitos), green (NHP), and aqua (cell cultures). **(B)** The total number of strains isolated per host species was used to derive the percentage of each host within the grand total.

The MR766 (HQ234498) strain of ZIKV from Uganda is considered the classical strain and is used consistently in both *in vitro* and *in vivo* research studies to model ZIKV infections. While this strain has been passaged 147 times in insect cell cultures and suckling-mouse brain tissues, very few mutations have been detected in its genome. In fact, when genomic sequences are compared between MR766 to two other variations, AY632535 and DQ859059, which were both isolated from Uganda in 1947 from sentinel Rhesus macaques, all three variants were determined to differ in only 0.4% of nucleotide and 0.6% of amino acid sequences (32). The initial low mutation rates may have been responsible for low transmission to humans and possibly subclinical infections. Phylogenetic analysis of available ZIKV genomes reveals that 86.5% of isolates are from humans, 11% are from mosquitoes, 2% are isolated from NHPs. Interestingly, of the available genomes, African lineages are only isolated from mosquitoes and NHPs, while Asian lineages are isolated from both humans and mosquitoes (Figure 1).

**Figure 2:**
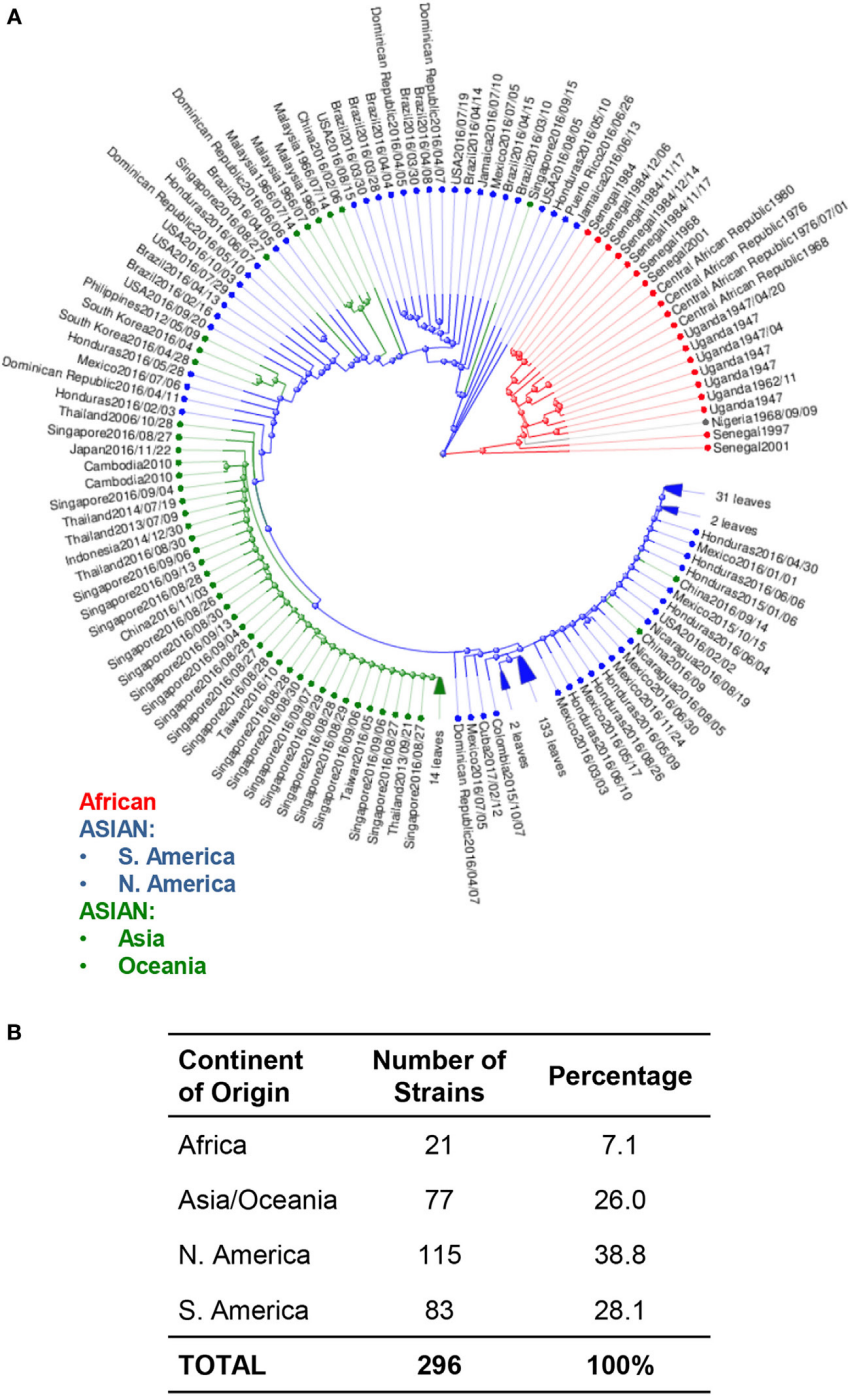
Phylogenetic analysis of Zika virus (ZIKV) genomes by region. **(A)** Phylogenetic analysis of available ZIKV genomes was organized according to lineage, followed by continent of isolation using Virus Variation analysis system available through NCBI. Only complete nucleotide genomes were screened and duplicate strains were removed to produce 296 unique strains. Strains were clustered from Africa (red), from the Americas (Asian lineage) (blue); and from Asia and Oceania (Asian lineage) (green). **(B)** The total number of strains isolated per continent was used to derive the percentage of each host within the grand total.

Phylogenetic trees of ZIKV have also been used to study the movement of ZIKV strains across the globe to identify potentially serious mutations that could alter molecular mechanisms, which then lead to enhanced pathology (33). Phylogenetic analysis of available ZIKV genomes reveals approximately 97% of genomes published are from the Asian lineage, and 7% are of African lineage. Among these Asian lineages, 66.9% of all isolates were collected from North, South, and Central America. Of these, 38.8% of isolates were from North and Central America, while only 28.1% were from South America. The remaining 26.1% of Asian lineage isolates were collected from the Asian and Oceania continents. Only 7% of all analyzed strains were from Africa (Figure 2).

A phylogenetic analysis juxtaposing MR766 strain to Asian lineage strains, particularly from Suriname and French Polynesia, reveals 50 amino acid lineage-specific differences (2, 34–37). Of these, all variations occur in either the NS1 or NS5 proteins (34, 35). Yet, when Wang et al. compared human to mosquito strains from the French Micronesia (FSM) outbreak in 2007 and the French Polynesia outbreak in 2013 (H/PF/2013), they identified 435 and 446 nucleotide changes in FSM and H/PF/2013, respectively, although 344 nucleotides were identical. Wang et al. considered them as sub-lineages deriving from the same ancestor that arrived in Malaysia in 1966 but had seemingly no clinical impact for 50 years (2, 26). All contemporary human strains within the Americas share higher sequence homology with the Asian lineage P6-740 (Malaysia/1966), which was the sole mosquito isolate (*A. aegypti*) than IbH-30656 (Nigeria/1968) (38). These isolates are most closely related to the H/PF/2013 strain (French Polynesia/2013) than the FSM strain (Micronesia/2007), suggesting that although the two Asian variants evolved from a common ancestor, they further diversified and the genetic distance between the 2007 and 2013 variants increased (2). A third major lineage from Africa is thought to exist based on analysis of only the E and NS5 gene sequences (35, 39). This lineage is designated Africa II, but it is neglected due to incomplete sequencing of the whole genome.

Multiple sequence alignments using 58 complete genome and five envelope sequences of ZIKV as of April 2016, revealed conserved amino acid variations. Nineteen variations were found in the sequence of structural proteins and 47 variations in the non-structural proteins, with the most variations in NS5 although the RNA-dependent polymerase domain had no variations at conserved motifs (26, 40). Eight variation sites were located in E protein; two sites were in the stem region and one site in the transmembrane region of the E protein. Substitutions in stem and transmembrane regions affect virion assembly and membrane fusion, whereas substitutions in Domain III of E protein may affect receptor binding (41, 42).

Smith et al. (27). found the difference between African and Asian isolates used in their analysis to be ~75–100 AA residues in the ORF, while the strains within each Asian and American lineage differed by ~10–30 AAs, suggesting that even minimal mutations could have phenotypic impact. A separate analysis by Wang et al. on nucleotide sequences compared 8 African strains (7 from mosquitos, 1 from monkey) with 25 Asian strains (all human) and found 59 amino acid variations that differed between the two major lineages, but were shared within the various strains of either African or Asian ancestry (2). The highest variability (10%) between Asian human and African mosquito strain was in the pr region of prM protein, though the effect of this structural change on viral function is not clear. Yuan et al. demonstrated differences in neurovirulence among Asian lineage strains from Cambodia and Venezuela may be dependent on a single amino acid substitution S139N (43). This substitution occurs in the pr region of prM and Yuan et al. hypothesizes that it may contribute to neurovirulent phenotype, but does not speculate as to a mechanism. These data are important because it demonstrates not only variances among strains of the same lineage but also sheds critical insight on intra-lineage strain-specific evolutionary differences. Comparison of protein sequences using P6-740 as the Asian reference and FSM showed over 400 variations at the nucleotide level and 26 unique substitutions at the protein level. Comparison of FSM, H/PF/2013, and the Brazilian strains from 2015 to 2016 showed that all these strains acquired changes at an additional eight positions for a total of 34 amino acid changes compared to P6-740. All isolates showed identical amino acids at these positions with the exception of T2634M/V in the NS5 protein.

Of note, no known ZIKV mosquito strain has the same nucleotide sequence as the human strains, though this could be due to sampling bias or ZIKV transmission through alternative routes (2). Nonetheless, nucleotide sequence changes can have an impact on viral pathology, replication, transmissibility, and fitness. One such example is the impact on posttranslational modification of the E protein. Faye et al. in 2014 reported a N154-glycosylation site deletion event of E protein in African isolates that did not exist in Asian lineage strains (40). Neurovirulence may depend on glycosylation of the Env protein (Asn 154) (44, 45). Naik and Wu reported that a mutation of putative N-glycosylation sites on DENV NS4B decreased RNA replication suggesting that glycosylation may play important roles in infectivity, maturation, and virulence of flaviviruses (46).

Similar to DENV, ZIKV evolution depends on worldwide spread of the mosquito vector, growing human population size, and increased foreign travel and commerce (37). Sequence analyses demonstrate that the virus originated in Africa within two distinct groups; Uganda and Nigeria, mostly isolated from non-human vectors, and anchored by the MR-766 strain. The Asian cluster was isolated in Malaysia and is anchored by a prototype strain, P6-740, which includes strains from other Southeast Asian countries, such as Cambodia and French Polynesia. The American clade, which includes strains from Brazil and other American or Caribbean strains, evolved from this Asian cluster and expanded rapidly among naïve populations (37). As ZIKV evolves, it diversifies and creates new interactions with vectors and hosts that impact pathology, which exhibit unique lineage and strain-specific pathological profiles. To this date, 197 fully sequenced African and Asian isolates have been characterized and have been deposited in GenBank.

## ZIKV Transmission and Tissue Tropism

### Vector Influence on Viral Evolution

While the African lineage contained eight mosquito isolates, the P6-740 (Malaysia/1966) was the sole mosquito isolate in the Asian lineage. In 2007, human sera from patients with painful febrile disease and *A. aldopictus* mosquitos were sampled from West Africa and tested positive for ZIKV (47). At the same time, the Micronesia outbreak identified *A. (stregomyia) hensilli* as the likely principal vector (6). In 2013, ZIKV reached French Polynesia, with subsequent spread to Oceanian islands (New Caledonia, Cook islands, and Easter island), which contained *A. aegypti* and *A. Aldopictus* throughout most of this region (48). Eleven percent of the population was infected causing symptoms such as low-grade fever, rash, conjunctivitis, and arthralgia, as well as GBS (49). *A. aegypti* has not only expanded to Central-South America but is also regarded as the most common vector for DENV (50). The New World strains of *A. aegypti* and *A. albopictus*, which are the most common in USA are poor transmitters of ZIKV (51) suggesting that continuous divergence of the Asian lineage due to genomic evolution can be adapted to direct human to human transmission without the involvement of a vector. Indeed, while *Aedes* is widely accepted as the vector for ZIKV (52–54), work by Guedes et al. has demonstrated that ZIKV can infect and replicate in the midgut, salivary glands, and can be detected in saliva of *Culex* spp. (55). This work suggests, while still a contentious topic requiring further investigation, the transmission vector range for ZIKV may be greater than anticipated.

### Non-Vector Transmission

Zika virus and other Flaviviruses (with the exception of Hepatitis C) are transmitted by mosquito bite, but ZIKV has clearly diverged from other flaviviruses. Since the 2015 epidemic, the mirth of published data has made it apparent that ZIKV can be transmitted from human to human through sexual transmission, blood transfusion, ocular transmission, or vertical transmission from mother to fetus (15, 16, 20). While the African strains are better transmitted through mosquitos, the American strains with Asian ancestry may have obtained enhanced transmission capabilities through sexual intercourse. This is supported by the numerous clinical cases of sexual transmission from male to female partners, and the limited data regarding female to male transmission (56–67). Of these clinical cases, originally infected individuals are reported to have traveled to South American countries or Pacific island nations, where they are believed to contracted ZIKV (68). Recent findings from Mead et al. reveal that while ZIKV can be detected in semen from infected men for up to 9 months after infection, sexual transmission of ZIKV typically occurs within 20 days of infection, and the amount of infectious ZIKV in semen decreases rapidly within the first 3 months of infection. Additionally, data from Barreto-Vieira et al. regarding attempts to standardize *in vitro* techniques for and Asian lineage strain of ZIKV demonstrated that mammalian cells associated with mucosal membranes are more susceptible to ZIKV infection than insect cells (69).

### Human Infection Studies

#### Fetoplacental Infections

The new routes of transmission demonstrate a novel tissue tropism for the virus. As such, tropism for the human placenta allowing infection of the fetus is unique for ZIKV, even though it should be noted that other flaviviruses that are not human pathogens do infect placental tissue in their respective hosts. Asian strains of ZIKV attracted global attention for their impact on maternal and fetal health (70, 71). Infectivity studies have shown that South American strains of Asian lineage are capable of infecting human decidua and umbilical cord tissues and are responsible for apoptosis of chorionic villi, which function in fetal/maternal blood/nutrient exchange (72, 73). In Brazil, mothers giving birth to newborns with microcephaly had reported fever, rash, and conjunctivitis during pregnancy, the most commons signs and symptoms of ZIKV infection. However, diagnosis beyond the earliest stage of acute disease is nearly impossible in the dengue-endemic regions and since the majority of exposures to ZIKV may cause asymptomatic infections, large numbers of infected pregnant women may have gone undiagnosed or misdiagnosed (20). Importantly, the severity of these responses varies greatly between patients and indicates that while studies on human immune responses to ZIKV infection are important, there is a large knowledge gap yet to be filled regarding the diversity of ZIKV infections among human demographics. An excellent example of how ZIKV pathogenic severity is dependent on individual genetic backgrounds is the study published by Caires-Junior et al., which compares pairs of twins where one is diagnosed with congenital Zika virus syndrome (CZS) (74). This study demonstrates that upon infection of neural tissues with ZIKV, cells from the CZS twins grew slower and exhibited increased viral replication. Importantly, the transcriptome results of the study reveal a significant difference in the level of an mTOR inhibitor protein, DDIT4L, between CZS and unaffected twins. This finding is significant because it indicates an individual genetic disposition for increased mTOR signaling, and as mTOR signaling pathways are critical for autophagy-mediate viral clearance, means that ZIKV infections are intensified in these individuals.

#### Fetal Brain Infections

Zika virus’s proclivity for neural cells is not a novel finding among flaviviruses, as members of this viral family are known for their neurovirulent qualities. Several studies have sought to investigate the neuroinvasive and pathogenesis of ZIKV within both human-specific cell cultures and in neonatal mice. One example is the work done by van den Pol et al., which investigates ZIKV cellular targeting within the brain (75). Here, *in vivo* mouse studies on neonatal brains reveals ZIKV infection of specific regions of the brain as early as 4 days postinfection, but can be fully determined by 7 days postinfection. Additionally, human cell culture work by van den Pol et al. reveals that human astrocytes demonstrate a 24-h virus incubation period before shedding active virus into culture media. Importantly, a study by Lin et al. using cultured human fetal brain tissues suggests that ZIKV enters the brain *via* the subventricular zone and actively infect and replicate in committed neural cells, and thus as cells propagate to develop the brain, result in an increase of viral presence (76). The authors then speculate as that the immune response to this neural infection may drive microcephaly.

#### Urogenital Tract Infections

Viral RNA can persist at high levels for months in the sperm of infected men even after resolution of symptoms and persists in the vaginal secretions of infected females for weeks after symptoms resolve (60, 77). This type of persistence in the reproductive tract and sexual transmission is not observed with other flaviviral infections (78).

#### Ocular Infections

Ocular infections are also unique to ZIKV and likely transmitted through conjunctival fluids, tears, and lacrimal glands. Although the reported cases of African lineage infections were too few to identify similar symptomatology, ocular infections by the Asian lineage have been documented in humans and recapitulated in animal models. Inner retinal vasculopathy (79) or other ocular infections have been reported linked to international travel into South American and Caribbean island nations (79, 80). Ocular abnormalities have been documented in infants with congenital Zika virus syndrome (CZS). In a report by Fernandez et al., postmortem examination of fetuses from terminated pregnancies revealed micro-calcifications of the retina, increased amount of autolysis of tissues at the front of the ocular tract, detachment of retinal and RPE layers, and a distinct lack of neural differentiation of retinal neurons (81). This study by Fernandez et al. corroborates with other clinical cases, such as those reported in Brazil, where 34.5% of all ZIKV microcephalic infants reported in the first 2 months of 2016 had ocular abnormalities, such as retinal mottling, optic nerve degeneration, and a lack of differentiation of retinal neurons (82–90).

### Animal Infection Studies

#### Ocular Infections

To investigate the pathogenic variability of ZIKV lineages as they occur progressively during active infections, many scientific investigations have modeled ZIKV infections in animal models. Noteworthy, among these are murine models, which have been well characterized for use in studying other flaviviruses. A129 and AG129 mice that are deficient in IFN receptor signaling when infected with ZIKV showed specific cellular tropism of ZIKV in retinal cell layers of the eye. A129 mice are deficient in IFN alpha/beta receptors, while AG129 mice are deficient in IFN alpha/beta and gamma receptors. Muller cells and retinal astrocytes infected with the virus resulted in a sustained proinflammatory microenvironment within the ocular tract that contributed to conjunctivitis and uveitis in these mice (91). In a NHP model for ocular and uteroplacental pathogenesis, Mohr et al. demonstrated lack of retinal neuron maturation, anterior segment dysgenesis, and notable chorioretinal lesions in fetal macaques (92). Different ZIKV strains display divergent tropism within host tissues. Miner et al. showed that ZIKV infections induced purulent viral panuveitis in AG129 mice and that the Asian lineage ZIKV from South America was isolated in higher viral burden than those from Oceania in ocular tissues (93).

#### Brain, Spleen, and Testicular Infections

Regarding tissue tropism by the Asian and African strains in animal models, the reports are conflicting. Dowall et al. demonstrated that viral RNA and ZIKV-induced histopathology with both MP1751 (African lineage) and PRVABC59 (Asian lineage) were highest in brain, spleen, and testis of A129, although the histological changes were more prominent in animals infected with the African lineage. The histopathology was minimal in heart, liver, kidney, and lung, although the Asian lineage caused no measurable clinical features (94). The Natal RGN strain from northeastern Brazil was isolated from the brain of a fetus with microcephaly and contained half of all mutations in the NS1 gene, suggesting that tissue-specific evolution of ZIKV has contributed to the emergence of Congenital Zika syndrome (2).

#### Lineage-Dependent Differences in Animal and Cell Culture Models

There are limited studies characterizing ZIKV strains and most studies utilize one strain exclusively. Phenotypic differences among African and Asian isolates have been reported in both *in vitro* and *in vivo* models, demonstrating the importance of considering ZIKV isolate, passage history, cell type, or mouse model when interpreting results. Asian strains of ZIKV have been analyzed for differential infectivity in many human and non-human cell lines. These include cells from ovary, kidney, liver, brain, lung, and keratinocytes. The latter of these is important to understand since the skin is the first barrier encountered during mosquito bites, as well as the first defense against ZIKV entry. Analyses from these *in vitro* studies demonstrate that infection after 48 h produced differences between cell lines in the amount of intracellular NS1, as well as amount of virus release, and the extent of infection did not directly correlate to IFN response (95).

Substantial differences are also found between African and Asian strains *in vitro* among mammalian and insect cell lines (27). A low-passage African isolate from mosquito reached higher titers than two low-passage Asian strains at all observed time points (0–36 dpi) in cell lines from four diverse vertebrate hosts and five insect cell lines (27). Similarly, Vielle et al reported strain-specific infection profiles in Vero cells, Aedes cells, and human monocytoid DCs (MoDCs). The authors used five African and Asian lineage strains isolated from various hosts; MR766 (U-1947) from monkey, MP1751 (U-1962) from a pool of *A. africanus* mosquito, PF13/25013-18: FP-2013 (French Polynesia) from human serum, PR-2015 (Puerto Rico) from human serum, and G-2016 (Guadaloupe) from human semen. The low passage U-1962 and U-1947 are very distant phylogenetically. The African lineage U-1962 and the Asian lineage PR-2015 showed highest rates of infection in Vero cells compared to the other strains. Similar findings for the U-1962 were observed in the mosquito cell line. In contrast, *in vitro studies* of human MoDCs showed similar susceptibility to infection, activation/maturation, expression of type I and III IFNs or cell death between lineages. The authors reported that NS5 of U-1962 showed polymorphism compared to the other strains of the study, but none of the residues were putative STAT2 binding residues, suggesting that the levels of expression of mutations were independent of mutations in the NS5 sequence of the U-1962 strain (96).

Additionally, infectivity studies comparing the two lineages revealed that African strains of ZIKV can infect human neural progenitor cells and produce both higher titers of progeny virus, and also induce higher levels of cellular apoptosis (34, 97, 98). A study by Hamel et al. demonstrated similar findings using human astrocyte cell cultures, where they found African ZIKV strain HD78788 can reach higher infectious titers 24 h postinfection of human astrocytes and also induces less innate antiviral gene transcription than Asian strain H/PF/2013 (99). This trend was further confirmed in human dendritic cells, which are one of the primary cell types naturally infected by ZIKV (96).

While mice may not naturally become infected by ZIKV, they can be used as models for pathogenesis, and similar reports of differential lineage-specific infection characteristics have been published regarding *in vivo* mouse experiments. Zhang et al. conducted a study juxtaposing an older Asian lineage strain from Cambodia, with a more contemporary American strain from Venezuela to investigate potential differences in neuro-virulence between the two (100). They found that compared to the Cambodia strain, neonatal mice infected with Venezuelan strain of ZIKV demonstrated more neuronal cell death, obliteration of oligodendrocyte development, and an increase in the amount of CD68 and Iba1 positive microglia/macrophages in brain tissues. On the other hand, Qian et al. reported that African and Asian lineages showed similar levels of brain-development disruption in an organoid development model, thus prompting further questions regarding further analyses within the Asian lineages (101). This change between Asian and American, or alternatively between pre-epidemic and epidemic stains of ZIKV, may be attributed to new mutations between the two (102). Indeed, sequencing data comparing over 20 strains of ZIKV reveals at least 15 amino acid changes between epidemic and pre-epidemic strains, as well as the generation of a 9 amino acid bulge, rather than an external loop structure at the 3-prime UTR region of the NS5 sequence.

Mouse background strain, transgenic line, and pharmacological manipulation in wild-type strains all produce variable results using the same dose and strain of ZIKV from either lineage. The animal model chosen is one potential complication of comparing ZIKV isolates, but so is *in vitro* and/or *in vivo* passage history of isolates. There is a tradeoff between passage number and viral fitness in either vertebrate or insect hosts. A study by Haddow et al. suggests that high passage number of traditional African strains, such as MR766, in both Vero cells and suckling mouse brains has resulted in a distinct loss of glycosylation sites, which may thus affect pathology in other organs (32). The pathology of a low-passage African isolate from mosquito and two low-passage Asian strains were compared *in vivo* using two mouse models, the A129 mouse (deficient in type-I interferon receptor, Ifnar1^−/−^) and the IFN-I antibody blockade mouse. The A129 mouse is commonly used to study ZIKV because the virus has been shown to infect cells by targeting human STAT2 to suppress IFN signaling, and it has been proposed that since it does not bind murine STAT2, it cannot infect mice unless the type I IFN receptor is knocked out or blocked (1). Of additional note, the majority of investigations that utilize ZIKV grow their viral stocks in Vero cells, which do not produce IFN type I in response to a viral infection, and thus allows them to be permissive to ZIKV infections (103, 104).

According to Smith et al., the African isolate caused more severe clinical pathology and lethality in both mouse models, suggesting enhanced virulence of the African strain compared to both Asian strains. Significant phenotypic differences were also observed between the two Asian strains (CPC-0740 and SV0127-14) used in the study; SV0127-14 produced 10- to 100-fold lower titers in all cell types compared to CPC-0740, and it produced only mild clinical symptoms and 10% mortality in Ifnar1^−/−^ mice versus 90% mortality with the more virulent CPC-0740 (27). The IFN-I antibody mouse model was far less susceptible than the Ifnar1^−/−^ model, producing zero mortality and no clinical symptoms with CPC-0740. The same result was found using a more recent Asian isolate from Puerto Rico, PRVABC59 in the IFN-I antibody blockade mouse (27).

Similar to previous studies comparing African and Asian strains, Dowall et al. reported that A129 mice tolerated infections with an Asian strain well, while an African strain was lethal, with morbidity and mortality worsening in a dose-dependent manner. Interestingly, although the Asian strain produced no clinical symptoms, viral RNA levels were detected in various tissues, including brain, spleen, lungs, and kidneys and viral burden was detected in secretions, albeit the magnitude and time course of the Asian and African strain differed, with detection levels produced earlier using African infections. Moreover, seroreactivity revealed detectable antibody responses in the Asian infected A129 mice despite no clinical signs of illness (1).

## Current Understanding of ZIKV and the Host Immune Response

**Figure 3:**
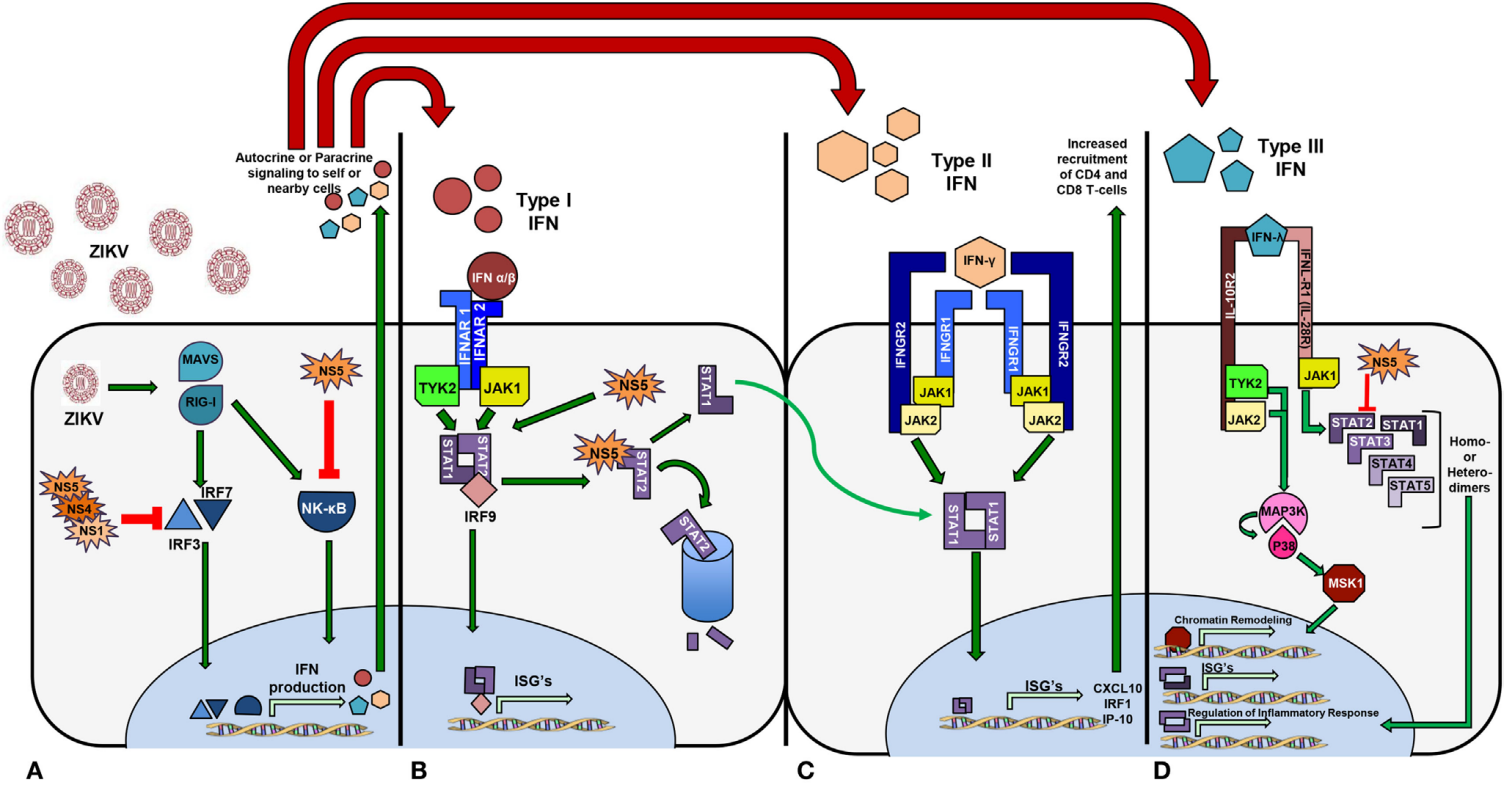
Zika virus activates signaling pathways that promote transcription of interferon (IFN) genes differentially. **(A)** The NS5 protein from African lineage binds and prevents NF-kB function. Asian lineage non-structural proteins NS5, NS4, and NS1 act to inhibit IRF3 and IRF7 functionality, thus inhibiting IFN production. **(B)** IFN Type I phosphorylates STAT1 and STAT2 and NS5 targets STAT2 for degradation. **(C)** Activation of Type II IFN signaling upon receptor binding phosphorylates STAT1 heterodimers and thus increases chemoattractant IFN-stimulated genes. **(D)** STAT2 is targeted for proteasomal degradation by NS5 resulting in an increased rate of STAT homodimerization and upregulation of anti-inflammatory cytokines.

Zika virus hid from the public eye for several decades since it’s discovery in the 1940s, as many cases of ZIKV infections are believed to have either been subclinical or misdiagnosed as a different flavivirus infection, such as DENV. As ZIKV spread from Africa across Asia and into the Polynesian and Micronesian islands, ZIKV shifted from a mild pathogenesis and largely subclinical symptomatology to the neuro-virulent Asian lineage with a higher incidence of congenital abnormalities. The specific pathways activated by viral infection inevitably steer the innate immune response toward differential patterns and intensities of cellular and humoral responses. ZIKV is a member of the flavivirus family, and thus shares similar cell signaling pathways with other viruses within this group, which directly antagonize the IFN response system of the host innate immune response, but through a species-specific mechanism (Figure 3). Thus, to fully comprehend the challenges that ZIKV poses to human immunity and to recognize fully efficacious vaccine candidates, a detailed understanding of how ZIKV directly evades and antagonizes host innate and adaptive responses is vital.

### Type I IFN Responses

Type I IFN refers to the classic IFN α/β signaling pathway, whereby viral antigen, or a pathogen-associated molecular patterns, are recognized by pathogen recognition receptors initiating an intracellular protein cascade that culminates in protein translocation into the nucleus and subsequent transcriptional activation of DNA for IFN-α and IFN-β (105). When nearby cells have IFN α/β bind to their surface receptor, IFN alpha receptor (IFNAR), a series of phosphorylation events, involving Janus kinase 1, tyrosine kinase 2, and the many signal transducer and activator of transcription (STAT) proteins ultimately result in the transcription of IFN-stimulated genes (ISGs), which have antiviral properties through a broad variety of mechanisms.

#### Lineage Similarities

Many flaviviruses directly antagonize different stages of the type I IFN pathway *via* species independent and diverse mechanisms that all centrally rely on genomic non-structural protein 5 (NS5). Of the seven genomic NS proteins and sub-proteins, the NS5 protein functions as the viral polymerase enzyme, and in RNA capping (106–108). Dengue virus NS5 protein has been shown to inhibit human STAT2 function by means of an E3 ubiquitin ligase, called UBR4 that targets STAT2 for proteasomal degradation. ZIKV similarly inhibits human STAT protein, but can do so independently of UBR4 (109, 110). ZIKV inhibition of STAT2 has been demonstrated in several studies where primary human dendritic or endothelial cell infections resulting in lower expression of pro-inflammatory cytokines, such as interleukin 6 (IL-6), IFN α/β, and chemokine C-C ligand 5 (CCL5) (111, 112). Additionally, NS1 and NS4b have both demonstrated the ability to inhibit the production of IFN α/β after direct stimulation with poly I:C (synthetic double stranded RNA) by blocking the formation of the TBK1 (TANK binding kinase 1) complex, which allows for oligomerization of interferon regulator factors (IRFs) (107, 113, 114).

Primary human skin fibroblasts infected with the French Polynesia isolate H/PF/2013 mounted innate immune responses by increasing the expression of RIG-I, MDA5, TLR3 leading to upregulation of Type I IFNs and ISGs as well as CXCL10 and CCL5 (115). The same strain led to IFN-β production and induced apoptosis of infected lung epithelial cells A549 (116). Overall, both lineages have demonstrated similar mechanisms of Type I IFN activation and upregulation, and similar pathways of STAT2 inhibition and targeting for degradation.

Importantly, ZIKV inhibits human STAT2 function *via* a mechanism similar to DENV, but does not similarly inhibit murine STAT2. These two proteins share 64% sequence homology, which may thus account for the dissimilar protein interactions. This single protein non-homology between human and mouse STAT2 has led to a domination of immune-deficient murine models as the primary model for pathogenic and vaccine studies (117, 118). These models are primarily A129 and AG129 systems, which lack the IFNAR protein, required for the production of ISGs. Despite many investigators’ claims that immune competent models for ZIKV will lack symptomatic expression, replication, and similarity to human infections, several recent publications have demonstrated that ZIKV can be modeled in immune competent mice using either a C57BL/6 or BALB/c background (119). The success of immune competent systems may be directly attributed to ZIKV inhibitory effects on STAT3, 4, 5, or 6. This knowledge, however, still requires investigation and remains a knowledge gap within the field.

#### Lineage Differences

Bowen et al. found that dendritic cells from donors are productively infected *in vitro* by both lineages, but with different kinetics. The African lineage had faster replication and infection magnitude, and unlike the Asian linage, caused cell death. All strains antagonized STAT1 and STAT2. Asian strains used included PRVABC59 and P6-740, while African strains included the Ugandan prototype strain (MR766) and a low-passage Senegalese strain (DakAr41524). They found that the viral kinetics varied with the source of the monocyte isolate, but that related strains varied similarly. All strains induced IFNB gene transcription, and a decrease in STAT2 phosphorylation was seen in both, but more pronounced in the African lineage (111).

In a study by Simonin et al. who infected human primary neural cells with African and Asian lineage strains, the African strain induced upregulation of at least 19 genes including RIG-I, MDA-5, and TLR-3 and induction of type 1 and 2 IFN was higher, associated with enhanced levels of inflammatory cytokines such as IL-6 or tumor necrosis factor (TNF). The only downregulated gene was CXCL8, a mediator of inflammatory responses. The Asian strain did not show any significant upregulation of genes; instead, four genes (CXCL8, CXCL10, CASP1, CTSS) were downregulated. Even at higher MOIs, the cytokine response to Asian strain was weak. In addition, neural cell infection by both strains showed similar differences in viral infectivity and cytokine production (97). In contrast, when McGrath et al. infected human neural stem cells from two individual patients with Asian and African lineages of ZIKV and conducted a transcriptomics analysis, they found increased expression of IFN-α, IL-2, TNF-α, IFN-γ genes as well as genes involved in complement, apoptosis, and STAT5 signaling pathways in cells infected with the Asian isolate (120). Patients displaying neurological symptoms during the ZIKV epidemic in Brazil demonstrated higher blood concentrations of pro-inflammatory cytokines, such as IL-6, IL-7, and IL-8, as well as higher levels of chemotactic molecules, such as IP-10 and MCP-1 (121). These findings were recapitulated in a non-human primate model (122) and in mouse studies modeling ZIKV infections using homologous strains.

Ultimately, this inhibition of type I IFN production and signaling results in an attenuated innate response to infection and may alter T cell-specific responses (123, 124). Presently, less than a handful of reports have examined the host-induced immune responses to genetically evolved ZIKV. Tripathi et al. examined strain-specific differences using Ifnar1^−/−^ and Stat2^−/−^ C57BL/6 mice. They looked at three viruses from the Asian lineage (P6-740, FSS13025, PRVABC59) and two from the African lineage (MR766; DakAr41519). They found that the African strains conferred faster onset of disease and higher mortality and in both mouse models. Infection with the African strains was marked by more severe neurological symptoms, while neurological symptoms in Asian infections were more prolonged. While both strains induced host inflammatory responses, the African isolates elicited higher levels of several cytokines and markers of T cell infiltration (IL-6, CXCL10, TNF-a, IFN-γ, CCL3, CCL4, CCL5, CXCL9, GZMB, CCL2, CCL7, CXCL1, CXCL2, IL-1b, IL-15, CD4, CD8, CCR5, CXCR3, CCR2, CCR5) (125).

Foo et al. infected human blood monocytes with the Uganda strain MR766 or the French Polynesia strain H/PF/2013. They found that both strains productively infected CD14 monocytes, but that infection with Asian viruses led to the expansion of non-classical monocytes, resulting in a M2-skewed immunosuppressive phenotype, marked by IL-10 production. African lineages on the other hand, induced pro-inflammatory M1-skewed responses, inducing CXCL10. They also found that blood from pregnant women was more susceptible to infection. Infection with virus from the African lineage led to higher viral burdens, and increased levels of IFN-β, STAT, OAS, IRF, and NF-kB. The Asian lineage had higher expansion of CD14^lo^CD16^+^ non-classical monocytes, despite having a lower viral load. In general, the African strain promoted cytokines and immunomodulatory genes involved with inflammation (CXCL10, IL-23A, CD64, CD80, IL-18, IDO, SOCS1, CCR7), while the Asian strain was associated with the activation of immunosuppressive genes (IL-10, Arg1, CD200R, CD163, CD23, CCL22, VEGFa). These results were confirmed using a second strain of ZIKV from each lineage (IbH30656 for African and PRVABC59 for Asian). Pregnancy enhanced infection of both lineages in CD14 monocytes, and they found a similar pattern with the African lineage having a higher viral load. Blood from the first and second semesters of pregnancy demonstrated considerably higher CD14^lo^CD16^+^ non-classical monocyte levels upon infection with the Asian strain, but blood in the third trimester had similar levels to non-pregnant blood. The African strain, however, produced a slight increase in non-classical monocytes during all three trimesters. Unlike monocytes from non-pregnant women, monocytes from pregnant women were more reactive to the Asian lineage than the African one. The Asian strain additionally induced genes associated with adverse pregnancy outcomes in the first two trimesters of pregnancy (ADAMTS9 and fibronectin 1) (73).

While both Asian and African lineages have demonstrated similar agonism and antagonism in Type I IFN signaling, Asian lineage strains have additionally shown a secondary method of IFN antagonism. Asian strains isolated from South American countries appear to directly activate IRF3, IRF7, and IRF9 through NS1, NS4, and NS5 viral proteins (112). African lineages have not demonstrated this ability as of yet, which implicates the differential amino acid residues as key binding factors for innate immune mediators.

### Type II IFN Responses

Unlike the type I IFN response, which inhibits ZIKV infection, Chaudhary et al. demonstrated that IFN-γ (a type II IFN) cannot only decrease ZIKV infectivity in mammalian cell culture, but can also upregulate transcription of innate inflammatory and chemoattractant cytokines, such as IRF1 and CXCL10, respectively. This suppression of type I responses, but activation of type II responses is also demonstrated by the NS2A and NS4B proteins. This phenomenon of variable activation of type I and type II IFN responses has also been documented in human clinical cases of ZIKV infections (126). A study performed by Kam et al., sought to fully characterize immune biomarkers that were associated with ZIKV infections in 95 human clinical cases from Brazil. The authors demonstrated an increase of IFN-γ among human febrile cases of ZIKV and differential cytokine expression between febrile patients and those with neurological complications. Between these two groups, patients with neurological complications showed similar levels of IFN-γ and decreased levels of anti-inflammatory cytokines, such as IL-10 and IP-10 (121). ZIKV NS5 was shown to generate the differential type I and type II responses during infection, by specifically inhibiting IFN-β signaling, and simultaneously functioning as a prominent activator of IFN-γ signaling.

Interferon-γ plays a critical role in host antiviral responses and increased levels of IFN-γ can be associated with host natural killer (NK) cell response, as they secrete high levels of this cytokine during infection. A study by V. Costa et al. proposed that DENV infection is controlled by NK cells specifically through the production of IFN-γ, and that these NK cells are activated by DENV-infected dendritic cells (DC’s) (127). While this specific mechanism of IFN-γ inhibition has not been demonstrated yet for ZIKV, it has been shown that ZIKV does infect antigen-presenting cells upon infection *via* mosquito bite. Cimini et al. found that the amount of IFN-γ secreted by CD4^+^ T-cells is reduced (128). Given that CD4^+^ and CD8^+^ T-cells have proven to be the dominant drivers in ZIKV clearance during human infection (129), this alteration in cell cytokine secretion generates several questions regarding the mechanisms that ZIKV uses to subvert the host immune response and facilitate its replication.

Ngono et al. compared CD8 T cell responses to two Zika strains from the African (MR766) and Asian (FSS13025) lineages in wild-type C57BL/6 mice treated with an IFNAR blocking antibody, and in LysMCre^+^IFNAR^fl/fl^ C57BL/6 (H-2^b^) mice (lacking IFNAR in certain myeloid cells). They found that the viral load of the African lineage strain decreased 3 days postinfection, but that the Asian lineage strain did not. Both strains elicited similar levels of granzyme B^+^ CD8^+^ T cells in both mouse models. They additionally identified epitopes recognized by IFN-γ secreting CD8^+^ T cells and found that in both strains the major epitope was E protein derived. In LysMCre^+^IFNAR^fl/fl^ mice, they identified 14 peptides specific to the African lineage, three specific to the Asian, and 12 shared by both, with all proteins being targeted, except for NS1 and NS2b in the Asian lineage. The Asian and African strains both resulted in a sixfold and fivefold increase in CD44^+^CD62L^−^CD8^+^ T cells, respectively, indicating a strong CD8 response. Both strains showed similar CD8^+^T cell kinetics, with the percentage of IFN-γ^+^ CD8^+^ T cells being highest at day 7 postinfection. It is important to note that the MR766 isolate was serially passaged in mouse brains, possibly affecting its behavior (124).

Collectively, the majority of the investigations regarding ZIKV and the Type II IFN response have been done using Asian lineage viruses. Thus, there is an immense knowledge gap concerning African lineage ZIKV strains and how they may directly affect the Type II IFN response. While it can be inferred that both African and Asian lineages both benefit from the increase in STAT1 being freely able to generate homodimers and thus promote ISGs, African lineage ZIKV strains have not specifically demonstrated this ability, and thus it remains unknown.

### Type III IFN Responses

Discovered in the early 2000s, type III IFNs comprises four variants of IFN-λ (numbered 1–4). The receptor for this type of IFN is unique because, rather than being ubiquitously expressed on nucleated cells like IFNARs, it is selectively expressed on epithelial cell surfaces. Thus, IFN-λ plays a distinct role in the protection of epithelial barriers. Additionally, while expressed on epithelia surfaces, other cell types can respond to type III IFN signaling, such as those in the central nervous system (130, 131). While the role of IFN-λ has been studied during WNV and YFV infections, there is limited information regarding IFN-λ and ZIKV infections. During DENV and YFV infection, the depletion of type III interferons results in impaired CD4^+^ and CD8^+^ T-cell activation, and thus also negatively impacts viral clearance. Additionally, in mouse models deficient for type III IFN signaling infected with YFV, type III IFN signaling results in decreased blood/brain barrier maintenance and thus allows for viral neuro-invasion (132). Of the studies for ZIKV infections investigating type III IFN responses to infection, many focus on maternal and fetal infections with emphasis on the fetal/maternal blood barrier (133–137).

The placenta is the organ that separates the fetal and maternal blood supply, primarily through the chorionic villi, where fetal and maternal blood are spatially separated by 3–4 cell layers. After blastocyst implantation in the uterine wall, trophoblast cells multiply and differentiate into variable cell types. One such cell type, the syncytiotrophoblast, forms the outer epithelial layer of the chorionic villi where the majority of fetal/maternal blood exchange occurs (138). The syncytiotrophoblast layer is the primary epithelial defense in the fetal/maternal blood barrier and the first cells ZIKV encounters during fetal infection. Indeed, the type III interferons produced by syncytiotrophoblasts allow for autocrine protection, and subsequently prevent ZIKV from infecting the fetus (139).

Provided that the cells of the fetal/maternal blood interface are resistant to ZIKV infections based on their ability to secrete IFN-λ, several studies have focused on uncovering the mechanism by which ZIKV gains entry into the amniotic space and thus can infect the fetus (140). These studies focused on a specific type of fetal macrophage cells called Hofbauer cells (HBC) that derive from the fetus; are of monocytic origin, and are commonly found through the chorionic villi (141). These cells first appear early during human pregnancy (within the first 3 weeks), and then diminish in number between the fourth and fifth month of gestation. Among mothers and fetuses infected with ZIKV, however, HBCs have been seen to linger long in to the third trimester of pregnancy at a density higher than normally observed (142). Not only do HBCs persist at increased density, they are capable of direct infection by ZIKV. It is speculated that they can move freely between the chorionic tissues where blood exchange occurs in the placenta (143–145). Thus, by traversing from the chorionic fetal/maternal blood interface to the amniotic sac and fetus, HBCs can act as a shuttle for ZIKV to bypass the fetal/maternal blood barrier and infect the fetus.

Asian lineage strains have shown the ability to upregulate Type III IFN production and mRNA translation in both cell culture and in primary human clinical cases. African lineage strains have only demonstrated this ability to a lesser extent only in cell cultures. *In vitro* studies with ZIKV AF (MR-766) and AS 9 (FSS13025) infected human choriocarcinoma JEG-3 cells showed induction of antiviral type III IFN responses and the ISG 2′-5′ oligoadenylate synthetase suggesting that IFN type III responses produced by human placental trophoblasts confer protection against ZIKV infection (143). Interestingly, while both lineages have demonstrated an increase in both translation and transcription, there is not an increase of the amount of active Type III IFN proteins produced and detected in culture medium.

### ZIKV Vaccine Development

Since the outbreaks that garnered international attention for ZIKV in 2015, the race for a vaccine to combat ZIKV has yielded several candidates, currently at various stages of development. A successful ZIKV vaccine must confer strong protection in healthy and pregnant populations, while also proving safe and efficacious in regards fetal/neonatal health. Important features to consider for the best vaccine should include both enhanced magnitude and quality of neutralizing antibodies and minimal cross-reactivity to DENV to prevent antibody-dependent enhancement (ADE) (146–149). ADE is perhaps the most critical of these, as flaviviruses are antigenically and structurally similar, non-neutralizing antibodies generated by one flavivirus can result in fatal outcomes upon secondary infection with a different flavivirus.

Recombinant envelope (E) protein subunit vaccines have been tested as both whole protein and single domain subunit candidates. Many of the E protein subunit vaccines published have required multiple booster administrations to achieve high antibody titers, and rely on the use of adjuvants to amplify the humoral response induced (150, 151). The use of only domain III of the E protein has proven sufficient to neutralize ZIKV in cell culture systems and in multiple mouse models when proteins are generated using the French Polynesian strain of ZIKV, Asian lineage, as a template (152). Combining the knowledge of how T-cells mediate viral clearance of ZIKV infections with epitope predictions, Pradhan et al. demonstrate that subunit vaccines using the NS2B, NS3, and NS4A proteins have potential as neutralizing vaccines that mediate a strong CD4^+^ T-cell response through *in silica* analyses (153).

Another candidate for ZIKV vaccines are lipid encapsulated mRNAs. While multiple publications have demonstrated that lipid enclosed mRNA vaccines are capable of antibody-based neutralization, only the work of Richner et al. address the issue of cross-reactive antibodies by specifically deleting the domain II of the fusion loop on the ZIKV E protein (154). Removal of this fusion loop domain results in minimal cross-reactivity between ZIKV and DENV, serotype 1. DNA vaccines have also been evaluated for ZIKV candidates, and one has progressed to clinical trials. DNA vaccines provide an economic advantage with their ease of production, and Larocca et al. has demonstrated a ZIKV DNA vaccine using the E and matrix (M) proteins can induce strong T-cell responses (155). An additional study by Muthumani et al. evaluated the efficacy of a DNA vaccine in both immune compromised mice and NHP and demonstrated the successful generation of neutralizing antibodies and high antibody titers (156). Of these vaccines, constructs generated as the vaccine have synthesized conserved residue among MR766 and Brazilian strains individually in an attempt to generate the largest cross-protective response. Despite this effort, data regarding non-homologous challenges and evaluations against different strains and lineages remains scarce. This is true for the majority of vaccine candidate studies published.

Live-attenuated vaccines and chimeric vector vaccines offer a third and fourth candidate against ZIKV, as they provide a strong cellular immune response due to their active infections, and thus provide a humoral response that more closely recapitulates a natural infection. Whole inactivated virus vaccines are the front-runner candidates for future ZIKV vaccines, based on the clinical trials occurring within the United States, 75% of which are whole inactivated particles from the PRVABC59 strain of ZIKV, which poses the largest threat to the US (157). Live-attenuated vaccines also have the benefit of generating a natural cellular response without the risk of a generating disease. Shan et al. has demonstrated that deletions within the 3′-UTR of the RNA genome can generate an attenuated ZIKV clone that fails to replicate, and that this vaccine can induce sufficient protection to prevent ZIKV from causing disease (158, 159).

### Knowledge Gaps and Future Studies

Despite the major concern that ADE and ZIKV antibody cross-reactivity among flavivirus plays in the development of vaccines, few studies successfully demonstrate that the neutralizing antibodies produced by their particular vaccine candidate are not cross-reactive to other flaviviruses. Thus, while the quest for neutralization is important, the future risk of ZIKV-mediated DENV ADE cannot and should not be overlooked, so that future DENV and CHIKV outbreaks can be avoided. A third consideration is that few, if any, publications directly investigate the binding avidity of these antibodies, but instead focus more on neutralization capacity, although the data strongly suggests low avidity and affinity antibodies have a higher chance of generating ADE for other flaviviruses, presenting another potential complication (152, 160, 161).

Additionally, despite the known disparities between different lineages of ZIKV, a lack of clarity remains regarding the mechanism behind neutralizing antibodies produced by a candidate vaccine or how it fairs against strains non-homologous to those in the vaccine. These neutralization assays should ideally include multiple strains from each of the three lineages: African, Asian, and American. This information is highly significant, because representative viruses from these lineages demonstrate differential neuroinvasive and inflammatory capabilities, as well as different infection profiles.

Few studies exist that focus on the impacts of ZIKV vaccination during pregnancy and even less address the issue of ZIKV sexual transmission. This is particularly important for areas with high prevalence of DENV where ZIKV cases can often be misdiagnosed and untreated for the infection. It is, therefore, of paramount importance to design effective vaccines conferring strong mucosal immunity to these high-risk groups.

## Conclusion

Zika virus continues to pose an international threat as both a neuroinvasive virus with potentially lethal consequences and as a direct danger to pregnant mothers. While ZIKV has demonstrated a continual geographic and phylogenetic expansion, the actual amount of genetic mutations is surprisingly limited between lineages. African lineages have shown to be more infectious and generate better inflammatory responses in a number of *in vitro* and *in vivo* experiments. The question that rises from the collection of studies that demonstrate a more virulent role for African strains is why the human impact, including neurological complications and fetal disruption, since 2015 in the Americas is produced by strains with Asian ancestry. It is also curious why the African strain is not found in any recently reported human cases. One possibility is that surveillance of infected humans in Africa is insufficient to detect severe cases of infection with the African strain.

Moreover, the African lineages exhibit an inhibitory mechanism on IFN production and signaling, which has been well documented in other flaviviruses. The Asian and American lineages, however, have evolved a secondary mechanism to prevent IFN transcription by IRF3 and IRF7 binding and preventing their translocation into the nucleus. Hence, while the African lineage has been shown to be more infectious than the Asian, it often presents as a self-limiting febrile disease, whereas the Asian and American lineages have exhibited persistent infections, with some human cases shedding active virus for upwards of 6 months (162).

Vaccine candidate research for ZIKV continues to be limited, providing little research into potential differences in vaccination responses between circulating lineages. Additionally, these investigations have not prioritized the critical relationship between ZIKV and other flaviviruses, such as DENV, as the antibodies proven protective against ZIKV may promote more severe infections for DENV, rather than provide cross-neutralizing benefits. While our understanding of ZIKV has increased profoundly, extensive investigations are still required for a better understanding of lineage-specific dynamics and the host immune response in terms of the evolutionary trajectory of these lineages as they continue to expand geographically. Improved understanding of these topics, which currently present knowledge gaps in the field of ZIKV research, will serve as the cornerstones for designing future vaccines and antivirals that not only efficacious for ZIKV, but also safe against other flaviviruses.

## Author Contributions

JB, NL, RH, and IS wrote and reviewed the manuscript, and IS approved the version to be published. All authors listed have made substantial and intellectual contribution to the work.

## Conflict of Interest Statement

The authors declare that the research was conducted in the absence of any commercial or financial relationships that could be construed as a potential conflict of interest.
